# Tactile estimation of hedonic and sensory properties during active touch: An electroencephalography study

**DOI:** 10.1111/ejn.16101

**Published:** 2023-07-30

**Authors:** Jessica Henderson, Tyler Mari, Danielle Hewitt, Alice Newton‐Fenner, Andrew Hopkinson, Timo Giesbrecht, Alan Marshall, Andrej Stancak, Nicholas Fallon

**Affiliations:** ^1^ School of Psychology University of Liverpool Liverpool UK; ^2^ Institute of Risk and Uncertainty University of Liverpool Liverpool UK; ^3^ Hopkinson Research Wirral UK; ^4^ Unilever, Research and Development, Port Sunlight Birkenhead UK; ^5^ Department of Electrical Engineering and Electronics University of Liverpool Liverpool UK

**Keywords:** EEG, estimation, hedonic preference, somatosensory, tactile, texture

## Abstract

Perceptual judgements about our physical environment are informed by somatosensory information. In real‐world exploration, this often involves dynamic hand movements to contact surfaces, termed active touch. The current study investigated cortical oscillatory changes during active exploration to inform the estimation of surface properties and hedonic preferences of two textured stimuli: smooth silk and rough hessian. A purpose‐built touch sensor quantified active touch, and oscillatory brain activity was recorded from 129‐channel electroencephalography. By fusing these data streams at a single trial level, oscillatory changes within the brain were examined while controlling for objective touch parameters (i.e., friction). Time–frequency analysis was used to quantify changes in cortical oscillatory activity in alpha (8–12 Hz) and beta (16–24 Hz) frequency bands. Results reproduce findings from our lab, whereby active exploration of rough textures increased alpha‐band event‐related desynchronisation in contralateral sensorimotor areas. Hedonic processing of less preferred textures resulted in an increase in temporoparietal beta‐band and frontal alpha‐band event‐related desynchronisation relative to most preferred textures, suggesting that higher order brain regions are involved in the hedonic processing of texture. Overall, the current study provides novel insight into the neural mechanisms underlying texture perception during active touch and how this process is influenced by cognitive tasks.

## Graphical abstract

The neural correlates of active touch exploration of two textured stimuli were examined. Touch parameters recorded from a six‐axis sensor were controlled for during the analysis of oscillatory changes recorded with EEG. Rough textures increased alpha‐band ERD in contralateral sensorimotor areas. Hedonic processing of less preferred textures increased temporoparietal beta‐band and frontal alpha‐band ERD, suggesting involvement of higher order brain regions.

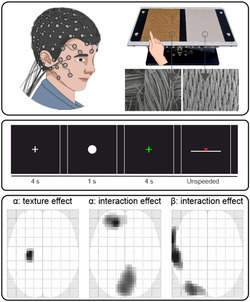

**Abbreviations**
ANOVA: analysis of variance
APC: aluminium composite panel
DLPFC: dorsolateral prefrontal cortex
EEG: electroencephalography
ERD: event‐related desynchronisation
ERSP: event‐related spectral perturbation
ERSP: event‐related synchronisation
GLM: general linear model
OFC: orbitofrontal cortex
SPM: statistical parametric mapping
VAS: visual analogue scale

## 1 INTRODUCTION

Humans encode somatosensory input to inform perceptual judgements. Texture is an important surface feature and is explored via the glabrous skin on the hands and digits via voluntary movement to create dynamic contact with surfaces, a behaviour termed active touch (Gibson, 1962; Prescott et al., 2011; Turvey & Carello, 2011; Wagner & Gibson, 2016). During tactile stimulation, subjective judgements of texture alter the blood‐oxygen‐level‐dependent signal, whereby greater prefrontal cortex activation was observed during tasks requiring estimation of surface properties, relative to conditions that included touch stimulation, but without estimation tasks (Kitada et al., 2005). In electroencephalography (EEG) literature, estimation tasks have been employed to quantify subjective judgements of surface texture (Ballesteros et al., 2009; Henderson et al., 2022), though ratings are typically collected separately from stimulation tasks. Therefore, it is unknown how subjective judgments modulate the electrophysiological correlates of texture processing.

Tactile information from surface texture is transduced by low‐threshold mechanoreceptors in the glabrous skin of the hands (Abraira & Ginty, 2013; Hagbarth & Vallbo, 1967; Johansson & Vallbo, 1979; McGlone & Reilly, 2010; Vallbo & Johansson, 1984). This information is conveyed to the primary and secondary somatosensory cortex via the dorsal‐column medial lemniscus pathway (Klingner et al., 2016; Raju & Tadi, 2021). Subsequently, the information can be transmitted to higher order regions involved in cognitive processing and multisensory integration (Gogolla, 2017; Morrison, 2016; Romanski, 2012; Uddin et al., 2017; Whitlock, 2017), where estimation of surface properties is more likely to occur.

Event‐related spectral perturbation (ERSP; Makeig, 1993; Makeig et al., 2004) is a spectral estimation method that provides insight into event‐related changes in the EEG spectra that are induced by the onset of stimuli (Grandchamp & Delorme, 2011). Decreases and increases in narrowband power are referred to as event‐related desynchronisation (ERD) and event‐related synchronisation (ERS), respectively (Pfurtscheller, 1977; Pfurtscheller & Aranibar, 1977, 1979). It is robustly shown that both motor and somatosensory activation are associated with ERD in alpha‐ (8–12 Hz) and beta‐band (16–24 Hz) frequencies, originating from the primary motor and somatosensory cortices, respectively (Brovelli et al., 2004; Pfurtscheller, 2001; Salmelin & Hari, 1994). Beta‐band ERS is then observed in the motor cortex after stimulation termination (Cheyne et al., 2003; Gaetz & Cheyne, 2006; Houdayer et al., 2006). Investigation of texture processing has revealed bilateral activation across sensorimotor areas, with an increase in cortical activation for smoother textures during passive touch (Genna et al., 2018; Moungou et al., 2016). On the other hand, active touch was found to elicit increased alpha‐band ERD for rough textures and increased beta‐band ERD for smooth textures (Henderson et al., 2022). Therefore, time–frequency analysis of induced cortical oscillations is an appropriate analysis method for active touch and is shown to elucidate bilateral sensorimotor activation associated with texture processing.

The prefrontal cortex is thought to be involved in cognitive control and executive processing (Menon & D'Esposito, 2021; Nejati et al., 2021) and has been identified as an important area during tactile discrimination tasks (Harada et al., 2004; Kitada et al., 2005; Marschallek et al., 2023; Stoeckel et al., 2003). The prefrontal cortex serves a well‐established role in the processing of affective value of stimuli (Rolls & Grabenhorst, 2008), including tactile stimuli delivered to glabrous skin (Francis et al., 1999; Rolls, O'Doherty, et al., 2003). Specifically, the dorsolateral prefrontal cortex (DLPFC) is active during somatosensory estimation and comparison tasks (Sathian et al., 2011; Simões‐Franklin et al., 2011; Yang et al., 2017) and is thought to reflect the storage of tactile information in working memory to later inform goal‐oriented motor behaviour (Barbey et al., 2013; Botvinick & An, 2009; Zhao, Zhou, et al., 2018). The orbitofrontal cortex (OFC) is associated with reward value and subjective pleasantness (Rolls, 2000, 2004), suggesting that prefrontal regions may also play an important role in hedonic preference for tactile stimuli, including surface texture (Gallace & Spence, 2011; Rolls, O'Doherty, et al., 2003). Research using EEG has linked frontal alpha‐band ERD to decision‐making (Ramsøy et al., 2018; Ravaja et al., 2013) and emotional valence (Al‐Nafjan et al., 2017; Poel et al., 2012; Schmidt & Trainor, 2010; Zhao, Zhang, & Ge, 2018). Further, beta‐band oscillations are thought to play a role in establishing a feed‐forward loop that connects somatosensory regions to higher order parietal and frontal brain regions (Adhikari et al., 2014). Taken together, research indicates that frontal alpha‐ and beta‐band ERD may be increased during somatosensory processing with estimation tasks, relative to tasks where no subjective estimation is required.

Alpha‐band oscillations are known to be modulated by attention (Klimesch, 2012; Pfurtscheller & Lopes da Silva, 1999). Cued attention during somatosensory tasks demonstrates decreased alpha‐band power in the primary somatosensory cortex (Jones et al., 2010; van Ede et al., 2011). In addition, attention increases beta‐band ERD prior to stimulus offset over sensorimotor areas (Bardouille et al., 2010; van Ede et al., 2011). Furthermore, the secondary somatosensory cortex is associated with tactile attention (Hämäläinen et al., 2000; Hoechstetter et al., 2000; Wu et al., 2014) and tactile discrimination (Kitada et al., 2005; Sathian et al., 2011; Stilla & Sathian, 2008). Estimation tasks are more likely to increase attentional demands, which may result in greater modulation of alpha‐ and beta‐band ERD in sensorimotor regions in contrast to tasks with no estimation.

The present study aimed to investigate cortical oscillatory changes during active touch exploration of rough and smooth textures during estimation and no‐estimation conditions. We hypothesised that active texture exploration would elicit bilateral alpha‐ and beta‐band ERD over sensorimotor regions. Specifically, we predicted increased beta‐band ERD for smooth when compared with rough textures, whereas alpha‐band ERD was hypothesised to increase for rough compared with smooth textures, following our previous research (Henderson et al., 2022). Further, we hypothesised that estimation tasks would result in increased sensorimotor and frontal ERD in alpha‐ and beta‐band, relative to no‐estimation conditions. Estimation tasks were split into two categories: sensory and hedonic estimations. We predicted that hedonic estimations would result in increased ERD in frontal regions when compared with sensory and no‐estimations conditions. Additionally, this study sought to investigate the potential differences between estimation type and texture by examining the interaction between the two variables.

## 2 MATERIALS AND METHOD

### 2.1 Participants

Thirty‐five right‐handed or ambidextrous participants were recruited (11 males, aged 18–49), left‐handed participants were excluded because of difficulty exploring the texture with their right hand. All participants had no history of any neurological condition, or aversion or allergies to any textures. Four participants were excluded because of excessive muscle artefacts or technical problems resulting in incomplete data recording from the touch sensor. The final sample for analysis included 31 participants (eight males, two ambidextrous), aged 28.13 ± 6.54 years. Participants were reimbursed at a rate of £10 per hour for their time. The study was approved by the Research Ethics Committee of the University of Liverpool, and all participants gave fully informed written consent at the start of the experiment in accordance with the Declaration of Helsinki.

### 2.2 Procedure

Participants were seated in a dimly lit Faraday cage with a 19‐in. LCD monitor approximately 1 m in front of them. The tactile exploration task and practice trials were presented using PsychoPy (Peirce et al., 2019). EEG and six‐axis sensor data were recorded during the tactile perception task. An arm support was used to stabilise and support the forearm while maintaining the position of the hand over the six‐axis sensor.

#### 2.2.1 Stimuli

**FIGURE 1:**
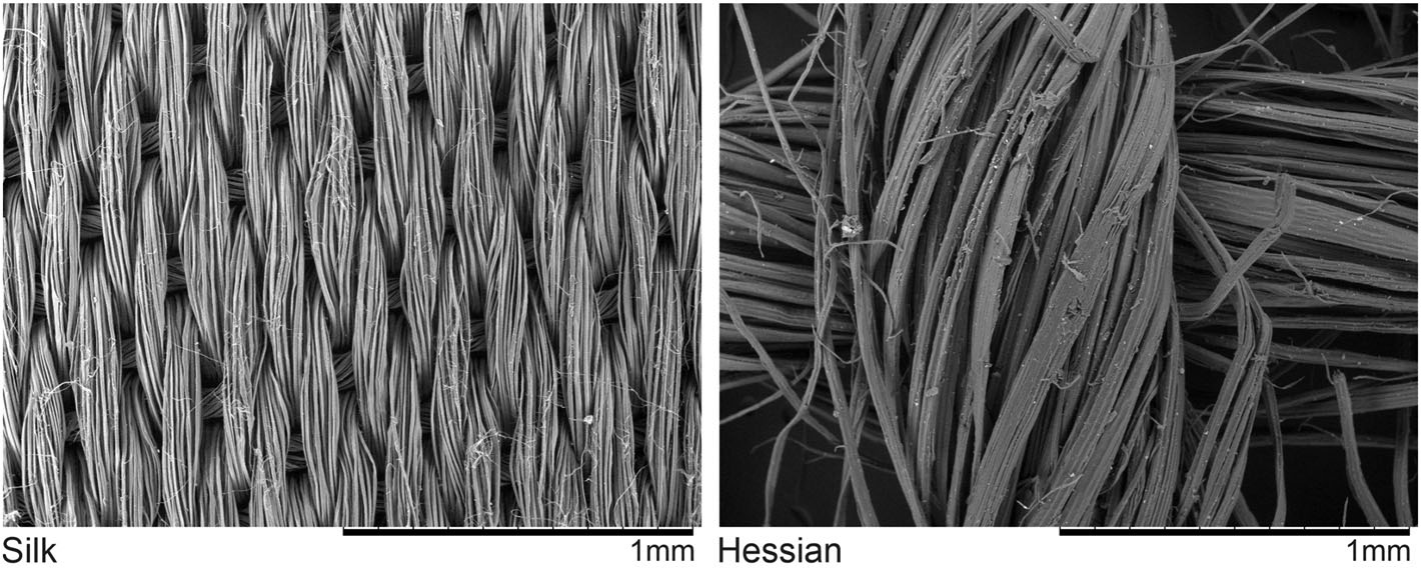
Hitachi TM‐1000 scanning electron microscope images of the texture stimuli at 100× magnification.

The stimuli included two textures selected from a previous study (Henderson et al., 2022): hessian and silk (Figure 1). The stimuli measured 150 × 255 mm and were mounted using double‐sided tape (tesa® 64621) to a paper sample mount measuring 410 × 255 mm lined with masking tape (tesa® Precision Mask® 4333). Samples were mounted in a portrait orientation; hessian was mounted on the left and silk on the right, with 40‐mm spaces on each side and a 30‐mm space between the samples. The paper sample mount was subsequently attached to an A3 size (420 × 300 × 3 mm) aluminium composite panel (ACP) secured to the Hopkinson Research six‐axis sensor (Hopkinson Research, 2020). The texture samples were replaced for each participant.

#### 2.2.2 Tactile exploration task

Participants completed four blocks, each lasting approximately 18 min. At the beginning of the block, participants were instructed to explore one of the two textures, and at the halfway point, participants were instructed to explore the other texture. A short break was given between blocks to increase task engagement and reduce desensitisation. There were 360 trials in total, 60 for each texture and each estimation condition (no estimation, sensory and hedonic). Each block consisted of 90 trials, including all three trial types; this task design aimed to maximise participant engagement by ensuring continuous attention to the condition indicators throughout the session. Block order was counter‐balanced, and the presentation order of trial conditions was pseudorandomised.

**FIGURE 2:**
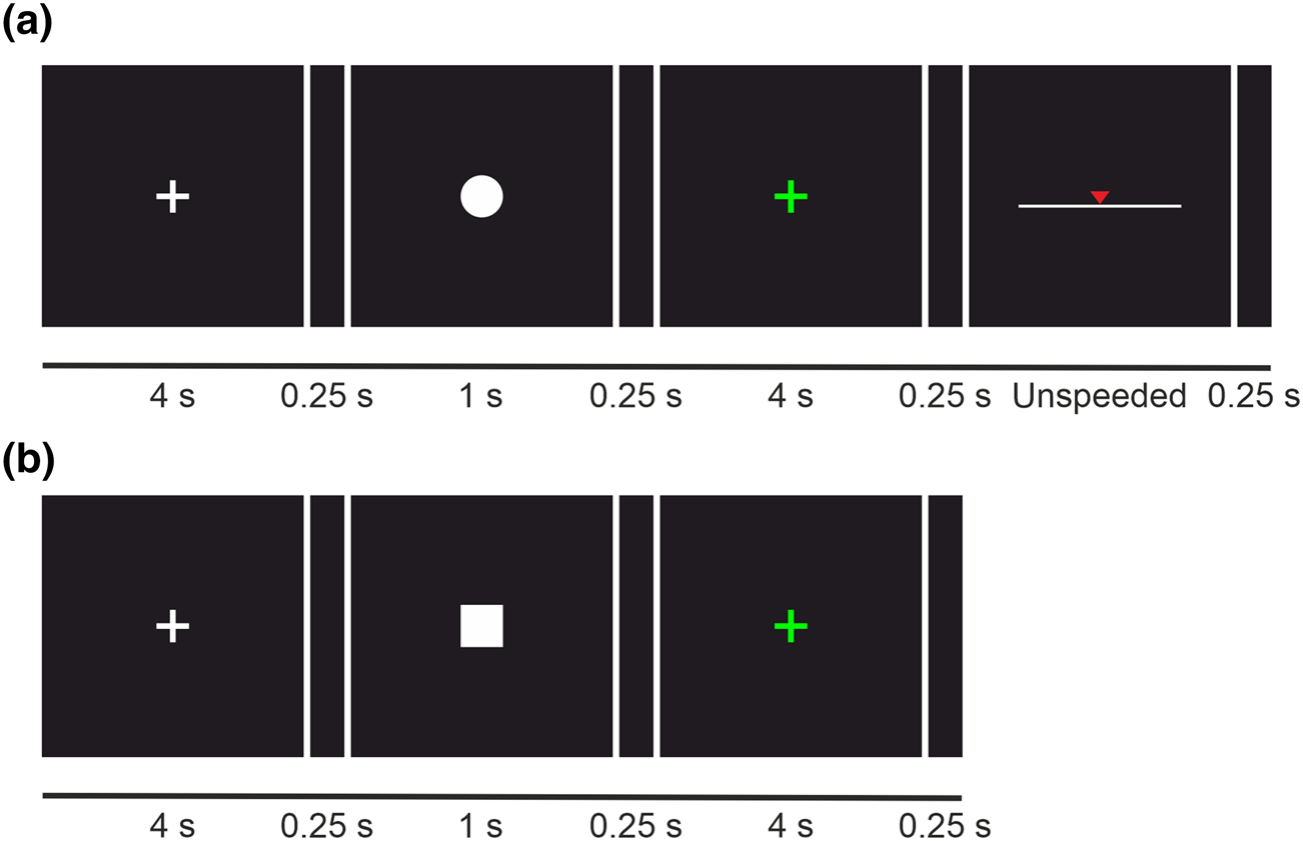
An example of a hedonic or sensory estimation trial (a) and a no‐estimation trial (a). For both conditions, each trial started with a baseline period indicated by a white fixation cross for 4 s, followed by the condition indicator for 1 s. Next, the tactile exploration period began, indicated by a green fixation cross presented for 4 s. For estimation trials, participants were instructed to rate the texture on the scale (i.e., smooth/rough or soft/hard for sensory trials, and pleasant/unpleasant or comfortable/uncomfortable for hedonic trials). In the no‐estimation task, participants did not perform ratings.

During the task, participants explored textures with the distal phalanx of their right index finger. Each trial consisted of a baseline period (4 s), condition indicator on screen (1 s) and tactile exploration (4 s), followed by an estimation response period for sensory and hedonic trials (Figure 2). The baseline period was indicated by a white fixation cross on the screen, during which participants kept their fingers stationary on the texture. The condition indicators, which were a white square, circle or triangle, were presented to specify whether the trial was sensory, hedonic or no estimation. Condition indicators were randomised between participants, meaning that each shape corresponded to each condition equally, thereby ensuring that the shape of the condition indicator did not influence the trial (Benikos et al., 2013).

Participants were trained to attend to sensory (i.e., ‘focus on how the texture feels. For example, is the texture smooth or rough, hard or soft?’) or hedonic features (i.e., ‘focus on how the texture makes you feel. For example, is the texture pleasant or unpleasant, comfortable or uncomfortable?’) during the respective trials. Subsequently, textures were rated on a visual analogue scale (VAS) corresponding to their respective trial type; for hedonic ratings, this included rating on pleasant/unpleasant and uncomfortable/comfortable scales, whereas sensory ratings included smooth/rough and soft/hard scales. During the no‐estimation trials, participants were not given any instruction to attend to any textural aspect and were not asked to evaluate the texture after the trial. Full task instructions and the VAS used are detailed in the Supporting Information.

During the touch exploration period, a green fixation cross appeared, indicating that the participant should start exploring the texture employing their preferred exploration behaviour, including multi‐directional movements as well as optimising their speed and load according to their preference. Exploration stopped when the green cross was removed from the screen. Participants were instructed to keep their fingers stationary on the texture when the green cross was not present (i.e., outside of the exploratory periods). Six practice trials following the same procedure were completed before beginning the tactile exploration task.

### 2.3 Recordings

**FIGURE 3:**
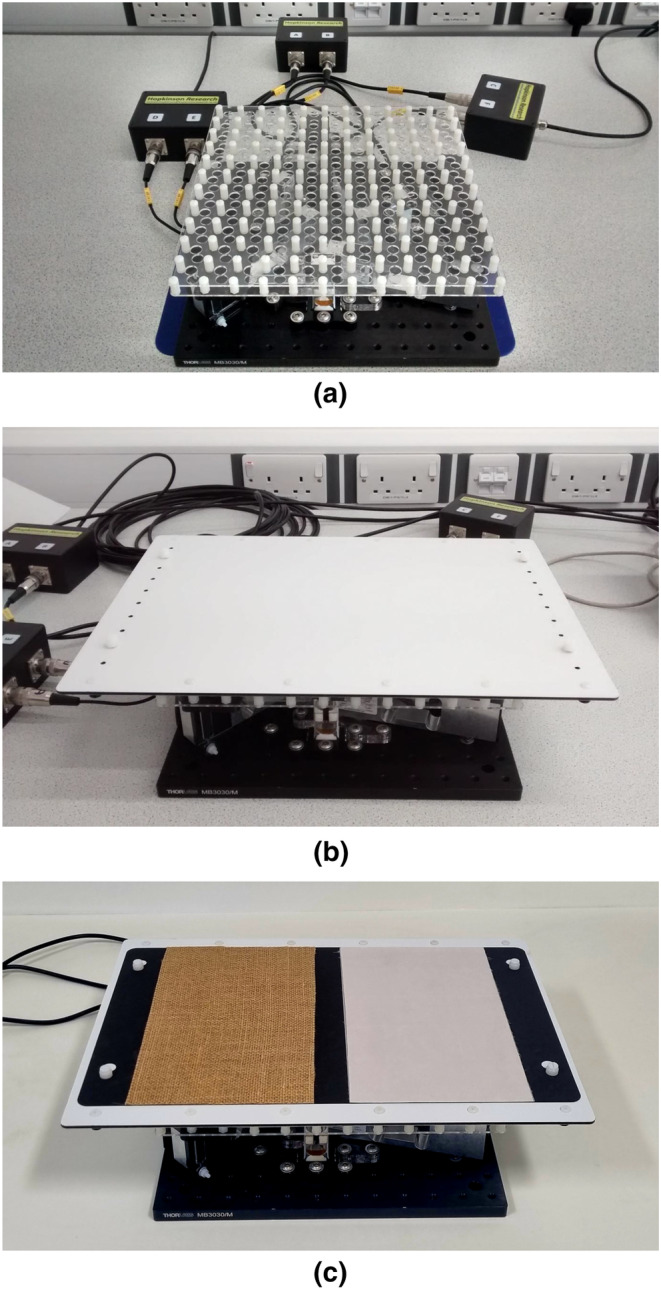
The setup of the six‐axis sensor with the load cells connected to the junction boxes (a) and with the aluminium composite panel (ACP) fitted (b). Textures stimuli are mounted to the paper sample mount and fixed to the ACP (c).

A 129‐channel saline‐based geodesic sensor net (Magstim EGI, UK) was used to record continuous EEG data. Positioning of the net was based on three anatomical landmarks, two preauricular points and the nasion. A recording band‐pass filter was set at 0.001–200 Hz with a sampling rate of 1000 Hz, and electrode impedances were kept below 50 k. Electrode Cz was used as a reference electrode and was not reinstated in the electrode array, leaving 128 recording channels. The six forces and torques acting on the texture samples due to the finger touch were recorded using the six‐axis sensor (Figure 3) with a sampling rate of 1000 Hz. Finger position and friction force in the XY plane and finger load along the Z axis were calculated from the block averaged (20 Hz) forces and torques. The speed of finger movement was calculated by determining the distance between two positions at different time points.

### 2.4 Pre‐processing

EEG pre‐processing was conducted using BESA v 6.1 (MEGIS GmbH, Germany). Eye blinks and electrocardiographic artefacts were removed using principal component analysis (Berg & Scherg, 1994). Data were filtered using 1‐Hz high‐pass and 100‐Hz low‐pass filters, with a 50 Hz ± 2 Hz notch filter. A visual inspection of data for the presence of any movement or muscle artefacts was performed, and trials affected by artefacts were excluded from subsequent analyses.

**FIGURE 4:**
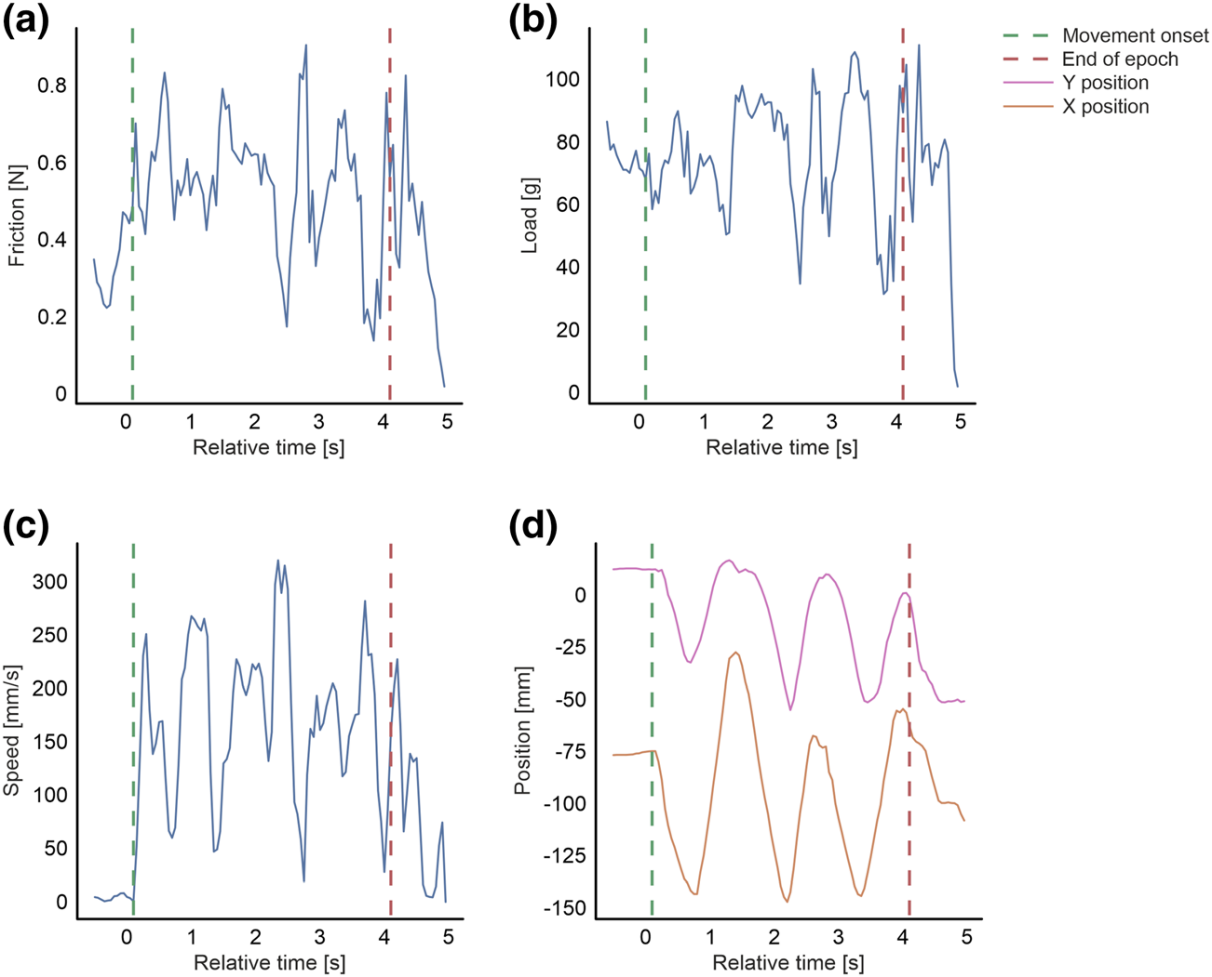
Line plots for one complete trial depict friction (a), load (b), speed (c), and *X*‐axis position in orange and *Y*‐axis position in pink (d). Dashed lines denote movement onset and the end of the epoch, which was the period used for the calculation of median parameters.

Six‐axis sensor data were cleaned and visually inspected using in‐house software developed in Python 3 (Van Rossum & Drake, 2009). Trials were rejected where no suitable triggers were identified, as an error during data recording. The trial period was epoched −5–5 s from visual trigger onset. Trials were rejected where movement was detected in the baseline or visual cue period. Data were then epoched −200–4000 ms relative to the trial onset cue. Movement onset was identified for trials by calculating and identifying the first minima and maxima peaks of velocity, with the height set at half the minimum and maximum value, and prominence and distance set to 1. Trials were rejected when movement onset did not occur within 400 ms of visual cue onset. Median speed, friction and load were calculated from movement onset to the end of the trial period. Figure 4 depicts a case example of one trial. *Z*‐scores were computed for each participant's measured touch behaviour during each block and texture. Trials were excluded if the *z*‐score of any measure exceeded ±2 standard deviations.

After completing EEG and six‐axis sensor pre‐processing, the average number of trials for analysis in each condition was as follows: sensory hessian, 39.52 ± 8.93 (M ± SD); hedonic hessian, 39.19 ± 9.35; no‐estimation hessian, 39.52 ± 8.88; sensory silk, 37.16 ± 8.84; hedonic silk, 39.39 ± 9.02; no‐estimation silk, 37.65 ± 8.70. The average number of accepted trials did not differ across conditions $\left(p>.05\right)$. EEG pre‐processing resulted in the rejection of 100.42 ± 47.97 trials over the entire experiment because of artefacts and noise. Furthermore, additional 23.42 ± 20.48 trials were rejected as a result of the six‐axis sensor pre‐processing.

### 2.5 Analysis

Event‐related time–frequency analysis was conducted using synchronised EEG and touch sensor data. Movement onset timings, computed on a trial‐by‐trial basis relative to the trial‐onset visual cue, were synchronised to EEG data. EEG data were epoched −5.5–5 s relative to the movement onset marker.

Data were imported into the SPM12 software package (Statistical Parametric Mapping, University College London, England) in MATLAB (The MathWorks, Inc., USA). Epochs were baseline corrected (−4 to −2 s). EEG signals were downsampled to 256 Hz and were re‐referenced using the common average method (Lehmann, 1984). Time–frequency analysis was performed by convolving the EEG signal with a set of complex Morlet wavelets, which are complex sine waves tapered by a Gaussian. The wavelets spanned a frequency range from 1 to 40 Hz with 1‐Hz steps, and each wavelet had 5 cycles (Cohen, 2019). The choice in cycle number ensured an acceptable trade‐off between time and frequency smoothing, with a slight preference for temporal precision (Cohen, 2019; Tallon‐Baudry & Bertrand, 1999). The power spectra obtained were rescaled with a log‐ratio transformation (−4 to −2 s), producing the baseline‐normalised ERSP. Subsequently, the power spectra were cropped from 0 to 4 s relative to movement onset and averaged over alpha (8–12 Hz) and beta frequency bands (16–24 Hz), giving narrow band ERD/S values.

For each trial, 3D scalp‐time images were generated by projecting the location of the 128 electrodes onto a 2D plane and then interpolating linearly between the point onto a standardised scalp grid sized 32 × 32 pixels (pixel size 4.25 × 5.38 mm^2^); the resulting topographies of power spectra planes are continuously stacked over each timepoint to give the 3D representation (X × Y × time). To address spatial and temporal variability between subjects and improve the conformity of images to the assumptions of random field theory, images were smoothed with a Gaussian kernel of 9 × 9 × 20 mm^2^ ms (full width at half maximum; Kilner et al., 2005; Worsley, 2007), as commonly utilised in previous research (Cook et al., 2015, 2017). SPM uses a technique called the summary statistic approach (Kiebel et al., 2007), where contrast images are generated in first‐level analyses to summarise the effects for each individual; subsequently, these images are utilised as data in second‐level models where the variability of the effects is assessed over the group of subjects.

First‐level analysis was conducted by applying the general linear model (GLM) to each subject's single‐trial scalp‐time data. The GLM design matrix consisted of six dummy variables specifying the trials texture (silk or hessian) and estimation condition (sensory estimation, hedonic estimation or no estimation) and six parametric regressors coding the summation of load and friction under the respective texture (silk and hessian) and estimation condition (sensory, hedonic or no estimation; e.g., summation of load and friction under sensory estimations of hessian); this can be seen in the Supporting Information. Regressors were mean centred to avoid multicollinearity issues. Three contrast images were produced to test for main effects: One image was produced to test the difference between hessian versus silk for the main effect of texture, and two images were produced to test the difference between sensory versus hedonic and hedonic versus no estimation for the main effect of estimation. Two contrast images were produced to test the interaction effect: the difference between sensory hessian and hedonic silk versus hedonic hessian and sensory silk and the difference between hedonic hessian and no estimation of silk versus no estimation of hessian and hedonic silk.

For the second level analysis, all 31 participants' individual contrast spectra from the first level were analysed using mass‐univariate analysis at the group level. The main effect of texture was tested using a one‐sampled *t*‐test and an *F*‐contrast of [1] with the 31 contrast images as input. A two‐sample *t*‐test design was employed to examine the main effect of estimation and the interaction effect. The 62 contrast images (two per subject) were entered, and an *F*‐contrast of [1 0; 0 1] was applied to test for these effects. An uncorrected cluster forming threshold of p<.001 and a cluster size of 35 contiguous space–time voxels, as commonly utilised in previous research (Cook et al., 2018), were used to determine significant effects. Power data from significant clusters were extracted and analysed in SPSS v. 28 (IBM Inc., USA) to determine the direction of observed effects: a paired sample *t*‐test for the main effect of texture, one‐way analysis of variance (ANOVA) for the main effect of texture and two‐way ANOVA for the interaction effect.

Mean subjective ratings of comfort and pleasantness (hedonic evaluations) and smoothness and softness (sensory evaluations) were calculated separately for each texture across all blocks. Subjective ratings were evaluated separately using 2 × 4 repeated measures ANOVA with two levels of texture (silk and hessian) and four levels of time (blocks one, two, three and four). Median speed (mm/s), friction (N) and load (g) were computed for each tactile epoch that was used in the EEG analysis. These data were analysed using a 2 × 3 repeated measures ANOVA with two levels of texture (hessian and silk) and three levels categorising the experimental condition (sensory estimation, hedonic estimation and no estimation) for each touch behaviour (speed, load and friction). Statistical outliers (±2 SD) were removed for all behavioural data.

The Greenhouse–Geisser epsilon correction was applied to all ANOVA analyses in cases where the data violated the assumptions of sphericity. Additionally, to account for multiple comparisons during post hoc analyses, the *α* level was adjusted using the Bonferroni correction.

Correlational analyses were performed on touch behaviour (speed, load and friction) for each texture (silk and hessian) independently. Factors found to be correlated were linearly combined by summation for subsequent SPM analysis to address issues of multicollinearity (Kalnins & Business, 2018). Further, touch behaviours showing no statistical differences between texture or estimation were not entered as regressors in the SPM analysis. Therefore, the linear combination of friction and load under each condition were used as regressors in the SPM model (see section 3.3 below). Additional correlational analyses were performed to examine the relationship between subjective ratings (comfort, pleasantness, softness and smoothness) and touch behaviour (speed, load and friction).

## 3 RESULTS

### 3.1 Subjective ratings

**FIGURE 5:**
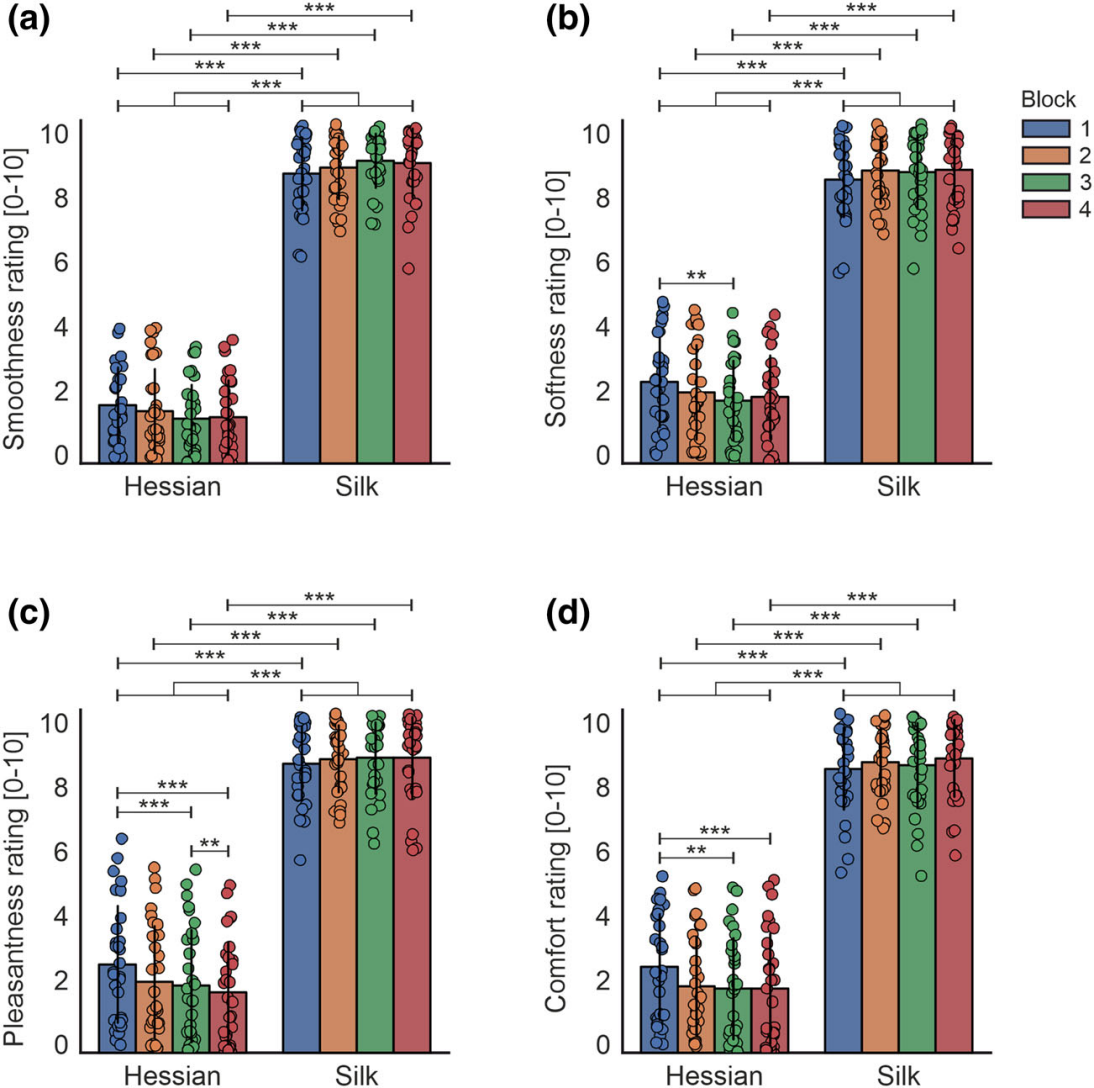
Bar charts showing mean subjective ratings, (a) smoothness rating, (b) softness rating, (c) pleasantness rating, and (d) comfort rating, for textured stimuli across experimental blocks. The individual dots show data points from each participant. Statistically significant differences are denoted as * for *p* < .05, ** for <.01 and *** for <.001.

Mean subjective ratings for each texture are shown in Figure 5. 2 × 4 ANOVA indicated statistically significant main effects of texture for ratings of comfort, $F\left(1,2479.56\right)=317.48,p<.001,\eta_{p}^{2}=0.93$; pleasantness, $F\left(1,2469.43\right)=263.49,p<.001,\eta_{p}^{2}=0.91$; smoothness, $F\left(1,2908.67\right)=610.11,p<.001,\eta_{p}^{2}=0.96$; and softness, $F\left(1,2306.62\right)=346.01,p<.001,\eta_{p}^{2}=0.93$. Pairwise comparisons revealed a reduction in comfort, pleasantness, smoothness and softness when comparing hessian to silk $\left(\mathrm{all}\;p<.001\right)$. Further, significant interactions between the effects of texture and time were identified for comfort, $\;F\left(2.17,3.44\right)=9.12,p<.001,\eta_{p}^{2}=0.27,\varepsilon =.72$; pleasantness, $\;F\left(2.08,5.22\right)=15.11,p<0.001,\eta_{p}^{2}=0.37,\varepsilon =.69$; smoothness, $\;F\left(1.90,1.85\right)=6.04,p=.005,\eta_{p}^{2}=0.19,\varepsilon =.63$; and softness, $F\left(1.73,2.94\right)=7.49,p=.002,\eta_{p}^{2}=0.22,\varepsilon =.58$.

The interaction between texture and time revealed that comfort, pleasantness, smoothness and softness ratings differed between exploration of hessian and silk when comparing against each respective block $\left(\mathrm{all}\;p<.001\right)$. Further, comfort ratings of hessian significantly decreased when comparing blocks 1 to 3 $\left(p=.003\right)$ and 4 $\left(p<.001\right)$. Pleasantness ratings for hessian decreased over time, with a significant difference between block 1 when compared with block 3 $\left(p<.001\right)$ and block 4 $\left(p<.001\right)$, and when comparing block 3 with block 4 $\left(p=.007\right)$. Softness ratings of hessian were significantly reduced when comparing block 1 with block 3 $\left(p=.005\right)$. By contrast, ratings of silk did not significantly differ across time for all ratings.

### 3.2 Touch behaviour

**FIGURE 6:**
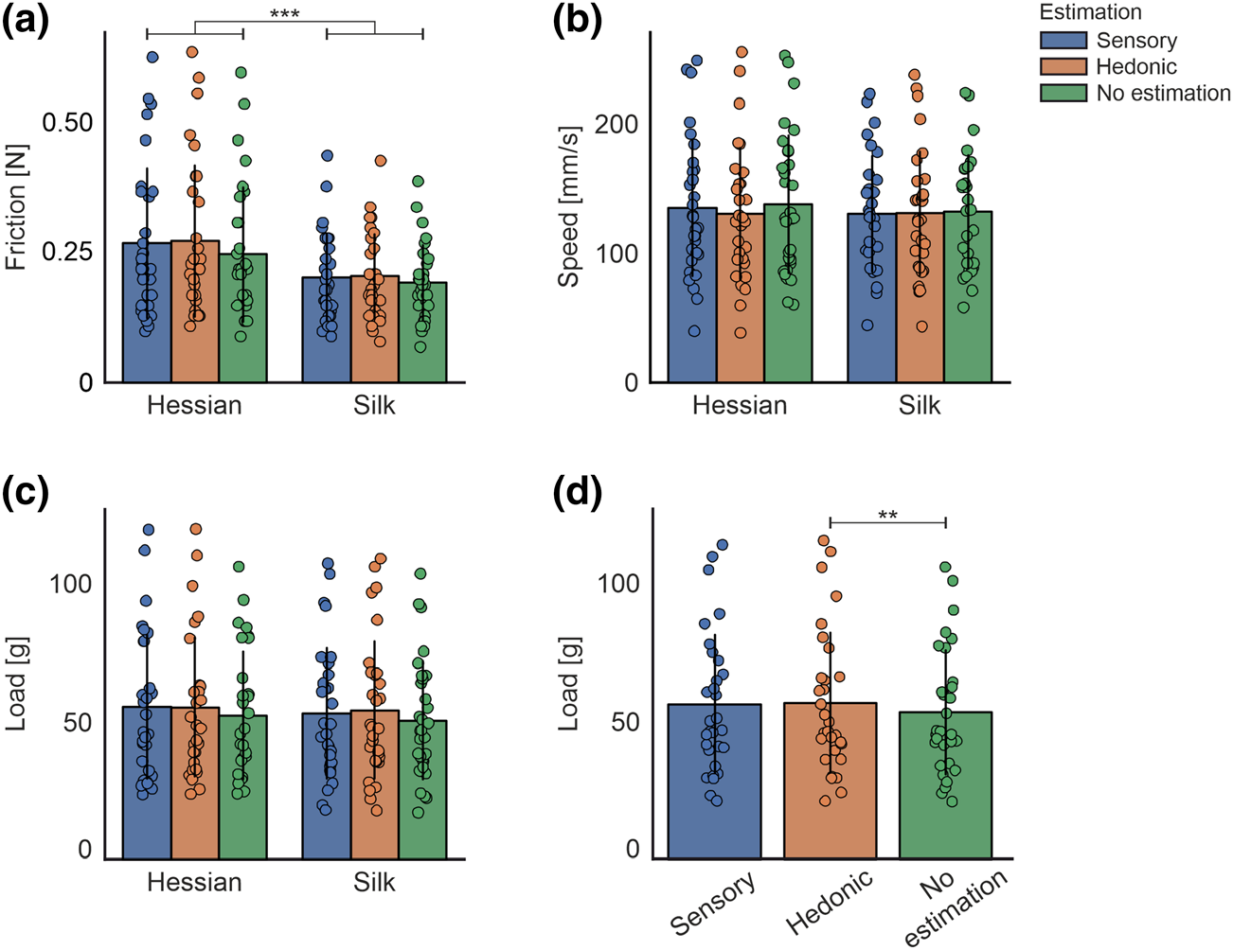
Bar charts showing mean touch behaviour, (a) friction, (b) speed, and (c) load, for textured stimuli across estimation trial type. (d) Bar chart showing mean touch behaviour for load averaged across textures to display the main effect of estimation. The individual dots show data points from each participant. Statistically significant differences are denoted as * for *p* < .05, ** for <.01 and *** for <.001.

Mean touch behaviour values of friction, speed and load for each texture are displayed in Figure 6. 2 × 3 repeated measures ANOVA, with two levels of texture and three levels of estimation, were conducted for each touch parameter. A significant main effect of texture was identified for friction $F\left(1,0.11\right)=15.54,p<.001,\eta_{p^{2}}=0.36$, and a significant main effect of estimation was demonstrated for load $F\left(2,72.55\right)=6.94,p=.002,\eta_{p^{2}}=0.20$. No significant differences were observed for speed. Pairwise comparisons revealed a significant reduction in friction (N) for silk compared with hessian $\left(p<.001\right)$ and a significant reduction in load (g) for no‐estimation trials compared with hedonic estimation conditions $\left(p=.001\right)$.

### 3.3 Correlational analyses

Correlational analyses performed on touch behaviour revealed a positive correlation between friction and load for exploration of hessian $r\left(29\right)=.86,p<.001$ and silk r(29=.75,p<.001. This was expected as friction force is directly proportional to load (Amontons, 1699; Blau, 2013). Therefore, friction and load were linearly combined by summation for inclusion in subsequent SPM analysis. Likewise, speed was not included as a covariate because of not differentiating between textures or rating types. Correlational analyses performed between subjective ratings, and touch behaviour revealed no significant correlation between any factors.

### 3.4 EEG

ERD/S was investigated relative to movement onset after accounting for the influence of load and friction on a single‐trial level. Group‐level analysis revealed significant scalp‐time clusters in alpha‐band, showing one cluster for the main effect of texture, one cluster for the main effect of estimation and two clusters for the interaction effect. In beta‐band, five clusters were identified for the main effect of estimation and two clusters for the interaction between texture and estimation type. No significant main effect of texture was revealed in beta‐band. The direction of observed effects was determined by subjecting power data, from significant clusters extracted from SPM12, to further statistical analysis in SPSS v. 28 (IBM Inc., USA).

#### 3.4.1 Alpha‐band

##### Main effects

**FIGURE 7:**
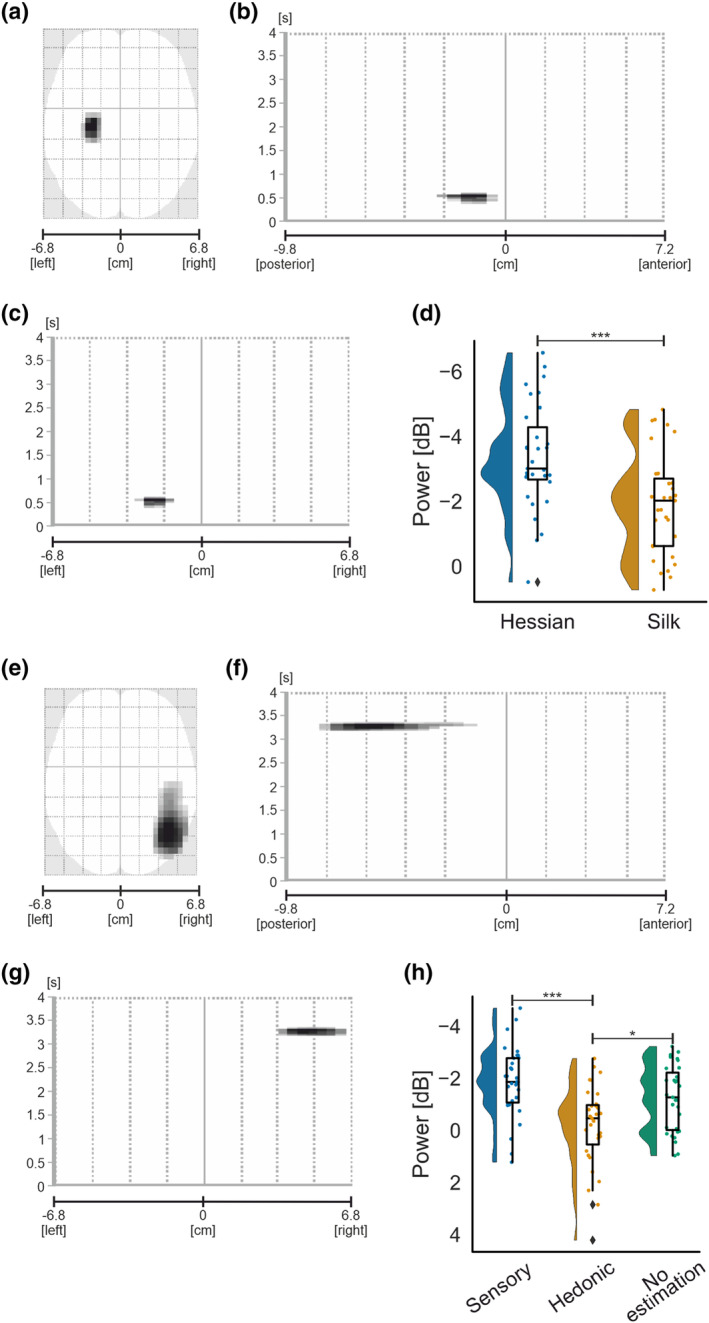
Standard scalp map of the statistically significant clusters in alpha‐band for the main effect of texture (a) and the main effect of estimation (e). Statistically significant latency periods 0–4 s relative to the onset of movement are displayed over the horizontal axis of the scalp (from left −6.8 cm to right 6.8 cm) for the main effect of texture (c) and estimation (g) and over the vertical axis of the scalp (from posterior −9.8 cm to anterior 7.2 cm) for the main effect of texture (b) and estimation (f). Raincloud plots (Allen et al., 2019) show the distribution of grand average alpha‐band power values for significant clusters for the main effect of texture (d) and the main effect of estimation (h). The half‐violin plots depict the probability distributions of the data. The individual dots show data points from each participant. The boxplots indicate the median, upper and lower quartiles, as well as the interquartile range (IQR) between the 25th and 75th percentile, and the whiskers represent scores outside of the IQR. Statistically significant differences are denoted as * for *p* < .05, ** for <.01 and *** for <.001.

A significant main effect of texture was identified in contralateral parietal regions, corresponding to left sensorimotor areas, spanning approximately 234‐ms duration and peaking at 357 and 436 ms after movement onset, illustrated in Figure 7a. A subsequent paired sample *t*‐test demonstrated significantly greater ERD for hessian $\left(-3.36\pm 1.64\;\mathrm{dB};M\pm \mathrm{SD}\right)$ when compared with silk $\left(-1.91\pm 1.58\;\mathrm{dB}\right)$, $t\left(30\right)=-4.92,p<.001$.

Over ipsilateral posterior parietal regions, as demonstrated in Figure 7e, a significant scalp‐time cluster demonstrated a main effect of estimation on induced power peaking at 3268 ms after movement onset and lasting for approximately 191 ms in duration, $F\left(2,23.13\right)=10.431,p<.001,\eta_{p}^{2}=0.26$. Pairwise comparisons revealed a significant decrease in ERD for hedonic $\left(-0.16\pm 1.58\;\mathrm{dB}\right)$ when compared with sensory $\left(-1.87\pm 1.35\;\mathrm{dB};p<.001\right)$ and no estimation $\left(-1.23\pm 1.25\;\mathrm{dB};p=.014\right)$.

Overall, findings demonstrate a significant effect of texture on contralateral parietal regions, with greater ERD for hessian than silk during active touch. In ipsilateral posterior parietal regions, a significant main effect of estimation was observed, with a decrease in ERD for hedonic compared with sensory and no estimation.

##### Interaction effects

**FIGURE 8:**
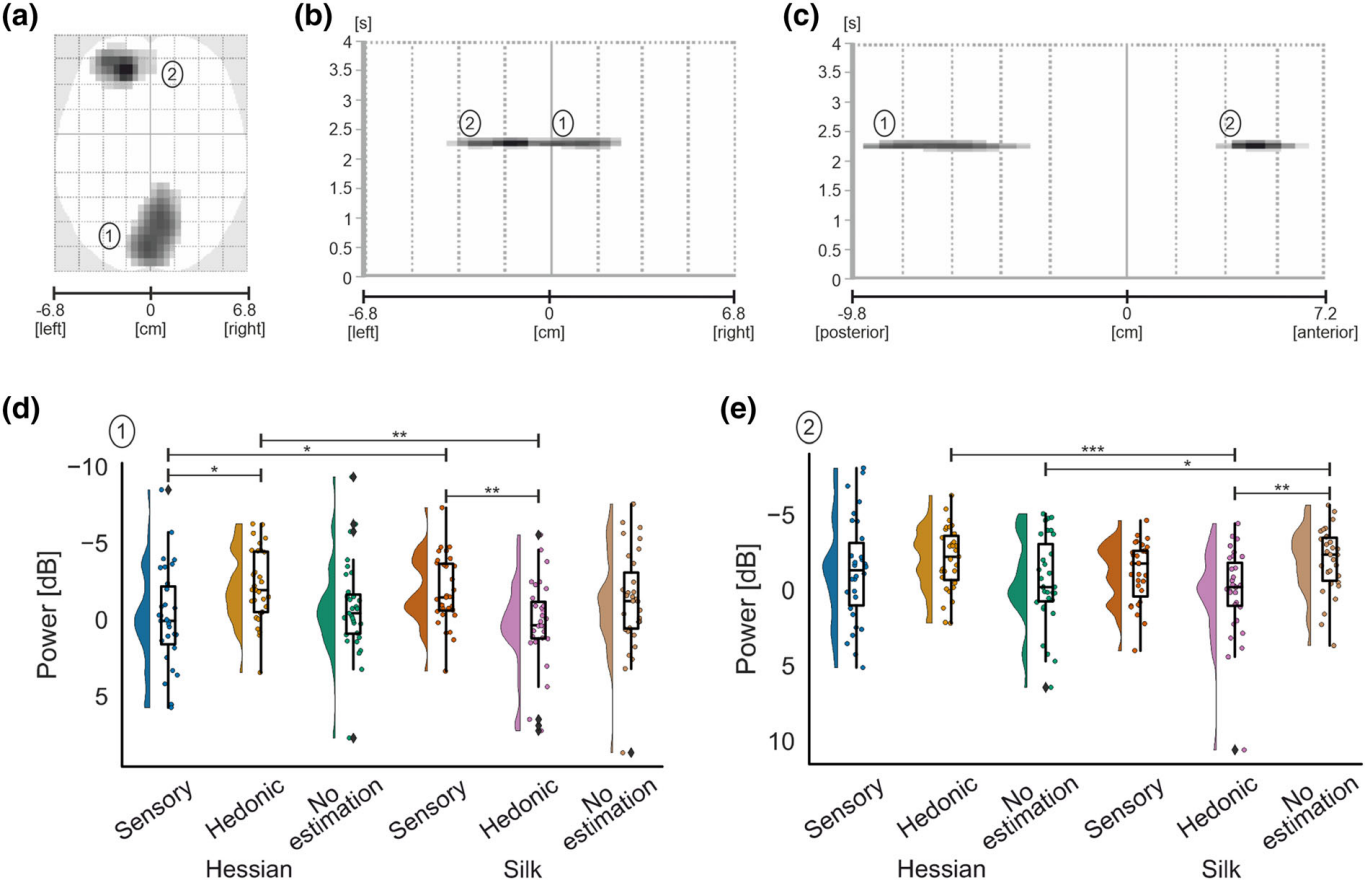
Standard scalp map of the statistically significant clusters in alpha‐band for the interaction effect between texture and estimation (a). Statistically significant latency periods 0–4 s relative to the onset of movement are displayed over the horizontal axis of the scalp (from left −6.8 cm to right 6.8 cm) (b) and over the vertical axis of the scalp (from posterior −9.8 cm to anterior 7.2 cm) (c). Raincloud plots (Allen et al., 2019) show the distribution of grand average alpha‐band power values for significant clusters for the interaction effect in cluster 1 (d) and cluster 2 (e). The half‐violin plots depict the probability distributions of the data. The individual dots show data points from each participant. The boxplots indicate the median, upper and lower quartiles, as well as the interquartile range (IQR) between the 25th and 75th percentile, and the whiskers represent scores outside of the IQR. Statistically significant differences are denoted as * for *p* < .05, ** for <.01 and *** for <.001.

An interaction between texture and estimation produced two significant clusters (Figure 8a). The largest cluster (*k* = 399) was located over bilateral occipital areas and encompassed approximately 173‐ms duration, peaking at 2213 ms, $F\left(2,73.91\right)=9.69,p<.001,\eta_{p}^{2}=0.24$. Exploration of hessian under hedonic estimation trials produced ERD, whereas exploration of silk produced slight ERS, resulting in a significant difference between the two conditions ( p=.002; Table 1). In addition, sensory estimations of silk produced ERD, leading to a significant difference between sensory and hedonic estimation of silk ( p=.002; Table 1). Sensory estimations demonstrated increased ERD for silk compared with hessian ( p=.021; Table 1. Further, greater ERD was observed for hedonic contrasted with sensory estimations for hessian ( p=.016; Table 1).

**TABLE 1:** Descriptive statistics for each significant cluster for the interaction effect in alpha‐band.

	Sensory				Hedonic				No estimation			
	Hessian		Silk		Hessian		Silk		Hessian		Silk	
Cluster	M	SD	M	SD	M	SD	M	SD	M	SD	M	SD
One	−0.08	3.34	−1.86	2.25	−2.17	2.36	0.32	3.00	−0.76	3.09	−1.20	3.32
Two	−1.29	3.41	−1.16	2.01	−2.08	2.13	0.14	2.92	−0.62	2.94	−1.89	2.18

Cluster 2 (*k* = 216) for the interaction effect peaked at 2232 ms and spanned approximately 173‐ms duration. The cluster was located contralaterally in the left hemisphere and encompassed lateral and medial frontal areas, $F\left(2,47.82\right)=10.75,p<.001,\eta_{p}^{2}=0.26$. An interaction between texture and estimation was uncovered in the hedonic estimation condition; specifically, ERD was observed for hessian and ERS for silk (*p* < .001; Table 1). Further, ERD was increased for silk when contrasting no estimation ( p=.006; Table 1) with hedonic estimations. No estimation resulted in increased ERD for silk compared with hessian (*p* = .025; Table 1).

In summary, a significant interaction effect of texture and estimation was identified for contralateral frontal and bilateral occipital regions. Notably, greater ERD was observed for hedonic estimations of hessian relative to hedonic estimations of silk in both clusters.

#### 3.4.2 Beta‐band

##### Main effects

**FIGURE 9:**
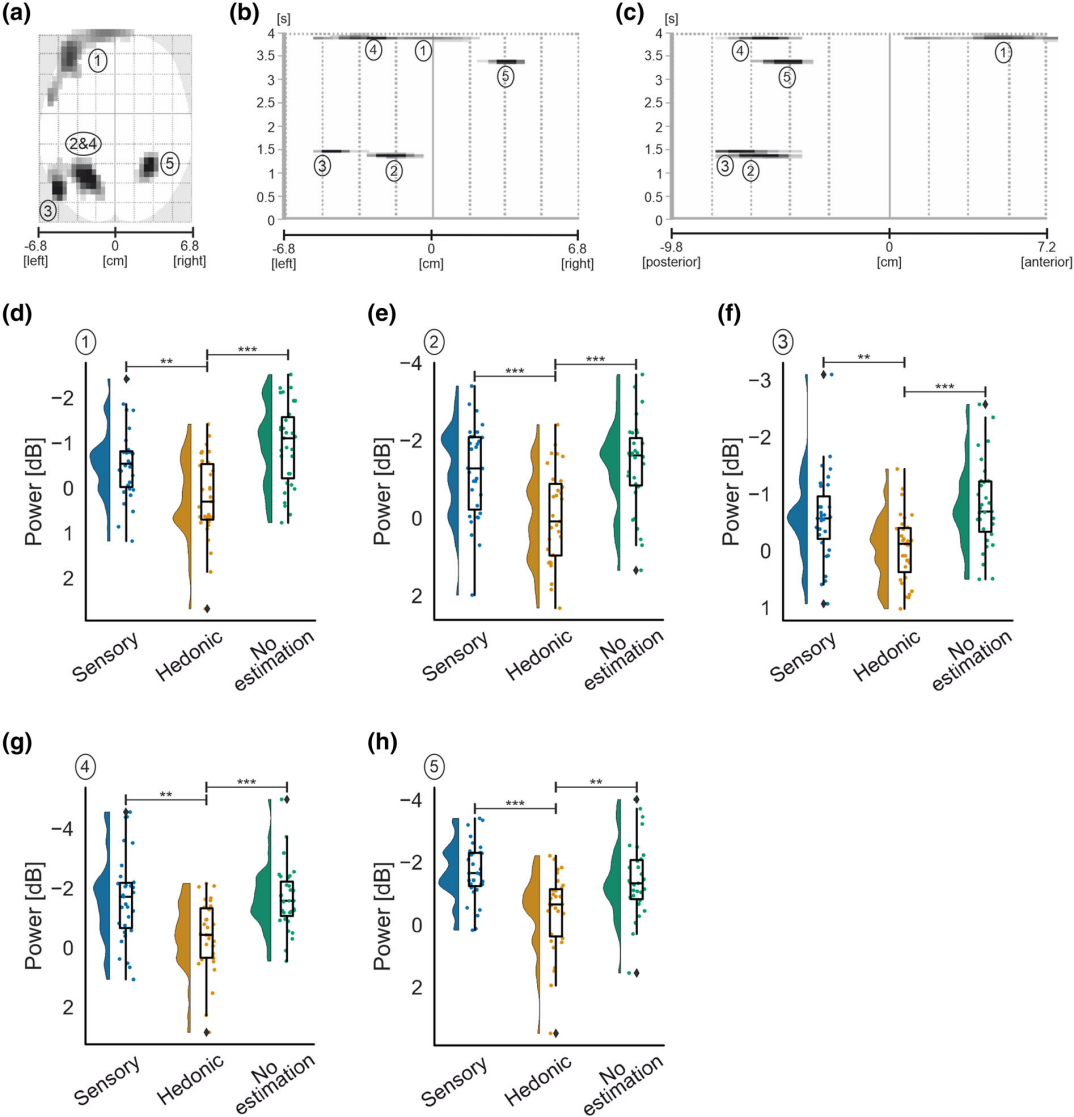
Standard scalp map of the statistically significant clusters in beta‐band for the main effect of estimation (a). Statistically significant latency periods 0–4 s relative to the onset of movement are displayed over the horizontal axis of the scalp (from left −6.8 cm to right 6.8 cm) (b) and over the vertical axis of the scalp (from posterior −9.8 cm to anterior 7.2 cm) (c). Raincloud plots (Allen et al., 2019) show the distribution of grand average beta‐band power values for significant clusters for the main effect of estimation in cluster 1 (d), cluster 2 (e), cluster 3 (f), cluster 4 (g) and cluster 5 (h). The half‐violin plots depict the probability distributions of the data. The individual dots show data points from each participant. The boxplots indicate the median, upper and lower quartiles, as well as the (IQR) between the 25th and 75th percentile, and the whiskers represent scores outside of the IQR. Statistically significant differences are denoted as * for *p* < .05, ** for <.01 and *** for <.001.

Five clusters were identified as statistically significant when investigating the main effect of estimation. The largest cluster (*k* = 181) spanned from contralateral frontal towards frontocentral areas (Figure 9a) peaking at 3873, 3893 and 3912 ms after movement onset and encompassing approximately 157‐ms duration. Subsequent one‐way ANOVA performed on EEG power data confirmed the significant main effect of estimation and tested the direction of observed effects (Table 2). Beta‐band ERD/S differences were observed between hedonic estimations, which elicited a marginal ERS response in this late period, and sensory and no estimations, which produced ERD. This led to significant differences when contrasting hedonic with sensory (*p* = .002; Table 2) and no‐estimation conditions (*p* < .001; Table 2). Cluster 2 (*k* = 69) and cluster 4 (*k* = 43) were located in contralateral posterior parietal regions and peaked at 1275 and 3893 ms while lasting approximately 95‐ and 138‐ms duration, respectively. Pairwise comparisons revealed a significant decrease in ERD for clusters 2 and 4 when comparing hedonic to sensory (cluster2:p=.001;cluster4:p=.002; Table 2) and no estimation (bothp<.001; Table 2).

**TABLE 2:** ANOVA and descriptive statistics for each significant cluster for the main effect of estimation in beta‐band.

					Sensory		Hedonic		No estimation	
Cluster	*df*	*F*	*p*	$\eta_{p}^{2}$	M	SD	M	SD	M	SD
One	2, 10.98	12.67	<.001	0.30	−0.53	0.78	0.22	0.92	−0.95	0.92
Two	2, 16.30	9.10	<.001	0.23	−1.19	1.21	−0.04	1.21	−1.38	1.17
Three	2, 4.65	8.10	<.001	0.21	−0.59	0.78	−0.05	0.6	−0.8	0.75
Four	2, 16.62	9.32	<.001	0.24	−1.64	1.46	−0.38	1.21	−1.66	1.1
Five	2, 14.50	8.88	<.001	0.23	−1.7	0.93	−0.41	1.26	−1.44	1.2

Cluster 3 (*k* = 44) was laterally adjacent to cluster 2 and lasted approximately 76‐ms duration, peaking at 1393 ms after movement onset. Pairwise comparisons demonstrated differences that were due to reduced ERD for hedonic estimations when compared with sensory (*p* = .004; Table 2) and no‐estimation (*p* < .001; Table 2) conditions. Finally, cluster 5 (*k* = 35) was ipsilateral to clusters 2 and 4 and peaked at 3365 ms, spanning approximately 77‐ms duration. Subsequent analysis revealed that the main effect of estimation was due to significantly decreased ERD for hedonic estimations relative to sensory (p<.001; Table 2) and no estimations (p=.006; Table 2).

The main effect of estimation for beta‐band can be summarised as eliciting a network of contralateral frontal and bilateral posterior parietal clusters, which all demonstrated a decrease in ERD for hedonic estimations relative to sensory and no‐estimation conditions.

##### Interaction effects

**FIGURE 10:**
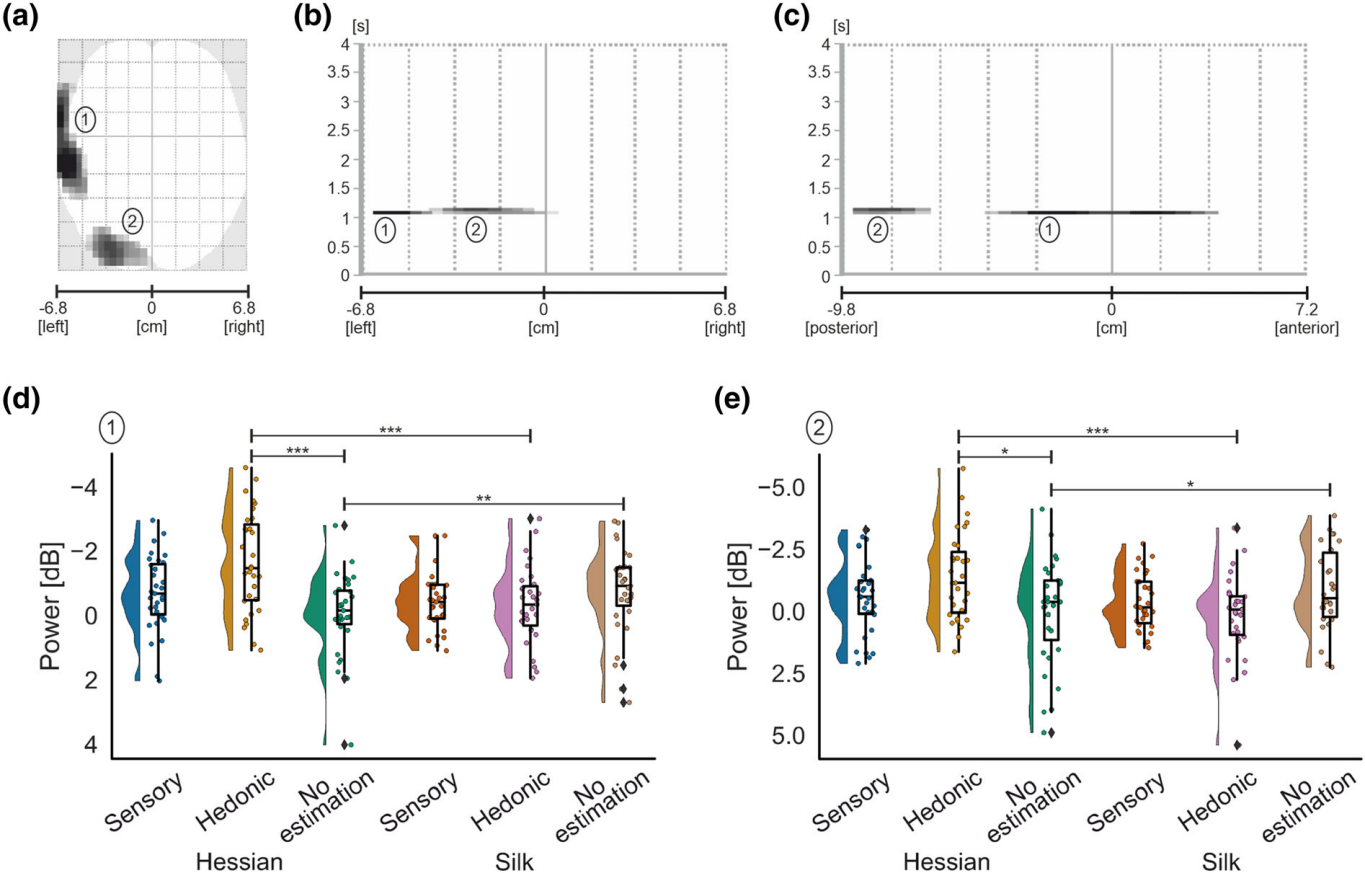
Standard scalp map of the statistically significant clusters in beta‐band for the interaction effect between texture and estimation (a). Statistically significant latency periods 0–4 s relative to the onset of movement are displayed over the horizontal axis of the scalp (from left −6.8 cm to right 6.8 cm) (b) and over the vertical axis of the scalp (from posterior −9.8 cm to anterior 7.2 cm) (c). Raincloud plots (Allen et al., 2019) show the distribution of grand average beta‐band power values for significant clusters for the interaction effect in cluster 1 (d) and cluster 2 (e). The half‐violin plots depict the probability distributions of the data. The individual dots show data points from each participant. The boxplots indicate the median, upper and lower quartiles, as well as the interquartile range (IQR) between the 25th and 75th percentile, and the whiskers represent scores outside of the IQR. Statistically significant differences are denoted as * for *p* < .05, ** for <.01 and *** for <.001.

Two significant clusters were identified for the interaction effect between texture and estimation (Figure 10a). The largest cluster (*k* = 94) was located over temporoparietal areas and spread to precentral regions, $F\left(2,16.14\right)=15.05,p<.001,\eta_{p}^{2}=0.33$. The cluster peaked at 963 and 982 ms and encompassed approximately 57‐ms duration. Increased ERD was observed for hessian compared with silk when exploring under hedonic estimation (*p* < .001; Table 3), whereas no estimation (*p* = .005; Table 3) produced greater ERD for silk compared with hessian. In addition, ERD increased during the hedonic estimation condition when compared with no estimations for hessian (p<.001; Table 3).

**TABLE 3:** Descriptive statistics for each significant cluster for the interaction effect in beta‐band.

	Sensory				Hedonic				No estimation			
	Hessian		Silk		Hessian		Silk		Hessian		Silk	
Cluster	M	SD	M	SD	M	SD	M	SD	M	SD	M	SD
One	−0.69	1.18	−0.45	0.94	−1.67	1.56	−0.33	1.19	−0.05	1.27	−0.75	1.36
Two	−0.6	1.53	−0.36	1.13	−1.41	1.77	0.2	1.66	0.01	2.12	−0.87	1.61

Cluster 2 (*k* = 76) for the interaction effect lasted for approximately 75 ms in duration and peaked at 1021 ms; the cluster was located over contralateral occipital areas, $F\left(2,23.95\right)=9.72,p<.001,\eta_{p}^{2}=0.24$. The interaction between texture and estimation revealed that ERD significantly increased for hedonic estimation of hessian compared with silk (*p* < .001; Table 3), whereas no estimation produced greater ERD for silk relative to hessian (*p* = .043; Table 3). Further, ERD increased for hedonic estimations compared with no estimations for hessian (*p* = .013; Table 3).

Overall, the interaction effect between texture and estimation revealed two clusters: one in contralateral temporoparietal regions and one in contralateral occipital regions. Notably, the results showed an increase in ERD for hedonic estimations of hessian relative to hedonic estimations of silk.

## 4 DISCUSSION

The purpose of this study was to examine oscillatory brain activity during active exploration of rough and smooth surface textures under conditions that necessitate the estimation of sensory or hedonic characteristics compared with no‐estimation conditions. Active tactile exploration with the index finger resulted in contralateral alpha‐band ERD over sensorimotor regions with greater ERD for rough (hessian) compared with smooth (silk) textures. Interestingly, hedonic estimations of hessian were associated with increased alpha‐band ERD in frontal and occipital regions and increased beta‐band ERD for temporoparietal and occipital regions, relative to hedonic estimations of silk. For the first time, touch behaviours were used as parametric regressors to investigate the effect of texture and estimation on neural responses while accounting for variation in touch behaviour at a single trial level.

Alpha‐band ERD was stronger in contralateral sensorimotor regions during exploration of rough hessian compared with smooth silk, consistent with previous findings (Henderson et al., 2022). Rough textures likely increase the firing rate of Merkel cells and Meissner corpuscles, which may produce greater alpha‐band ERD (Cascio & Sathian, 2001; Gamzu & Ahissar, 2001; Johansson & Vallbo, 1979; Vallbo et al., 1995). Further, the cluster peaked within a few hundred milliseconds of movement onset, suggesting the first neural response to the processing of physical attributes such as roughness (Ballesteros et al., 2009; McComas & Cupido, 1999). Previous passive touch research reported that smoother textures increase cortical activation (Ballesteros et al., 2009; Genna et al., 2018; Moungou et al., 2016), suggesting that activation in response to rough and smooth textures may differ depending on the mode of tactile stimulation. Future research should investigate active and passive modes of stimulation while manipulating surface roughness.

Sensory and no‐estimation conditions showed increased alpha‐ and beta‐band ERD relative to hedonic estimations. Alpha‐band ERD manifested as an ipsilateral posterior parietal cluster, an area associated with sensory integration (Hyvärinen, 1982; Mountcastle et al., 1975), whereas beta‐band ERD demonstrated a network of contralateral frontal and bilateral posterior parietal regions. Alpha‐band ERD in parietal regions enhances processing of task‐relevant sensory information (Klimesch et al., 2007; Pfurtscheller & Klimesch, 1991), whereas beta‐band connects somatosensory regions to higher order parietal and frontal regions (Adhikari et al., 2014). Contrary to our hypothesis, hedonic estimations generally demonstrated a decrease in ERD relative to sensory and no‐estimation conditions. Positivity and negativity are proposed to serve as bipolar opposites, implying that an increase in one dimension corresponds to a decrease in the other (Becker et al., 2019; Wundt, 1897). A fMRI meta‐analysis suggests that regions of the prefrontal and anterior cingulate cortex demonstrate dissimilarity in concordant activation to positive and negative affect, supporting bipolarity in regions of the brain (Lindquist et al., 2016). Pleasant and unpleasant stimuli may result in differential changes in ERD/S, which cancel out activation when averaging trials across both types of hedonic estimations (negative/positive). The interaction effect between texture and estimation type supports this hypothesis, as differences were observed between hedonic estimations of hessian and silk in frontal, temporoparietal and occipital regions.

Hedonic estimations of hessian elicited significantly greater ERD than silk in contralateral temporoparietal beta‐band. Decreases in beta‐band power are associated with increased subjective preference for food, face, olfactory, auditory and thermal stimuli (Bauer et al., 2015; Son & Chun, 2018; Tashiro et al., 2019; Yuan & Liu, 2022). In temporoparietal regions, beta‐band power distinguishes pleasant from unpleasant tactile stimuli during passive stimulation of the hairy skin, where the least preferred texture elicited greater ERD than more preferred textures (Singh et al., 2014). Further, ERPs elicited in response to rough tactile gratings were found to originate from the insular cortex (Ballesteros et al., 2009), a region tightly linked with the processing of hedonic preference (Morrison, 2016; Perini et al., 2015). Increased temporoparietal beta‐band ERD during hedonic ratings of hessian may be due to the activation of higher order somatosensory association regions, with the insula potentially playing a role in distinguishing the hedonic value of perceived unpleasant tactile stimuli.

Activation of visual areas by tactile stimulation with textured surfaces suggests the role of the visual cortex in integrating visuo‐haptic information to facilitate texture perception (Eck et al., 2013, 2016; Merabet et al., 2007; O'Callaghan et al., 2018; Sathian, 2016; Sathian et al., 2011; Simões‐Franklin et al., 2011; Stilla & Sathian, 2008). The textured stimuli were visible to participants in this study, suggesting that alpha‐ and beta‐band ERD in occipital areas reflects cross‐modal visuo‐haptic processing. Previous fMRI investigations of haptic texture processing demonstrate that estimation of surface roughness activates visual areas (Eck et al., 2013, 2016; Sathian et al., 2011; Stilla & Sathian, 2008), and visual texture modulates pleasantness ratings of haptically explored textures (Etzi et al., 2018). Therefore, increased occipital ERD during hedonic estimation of rough textures may reflect greater reliance on visual information than haptic information in sensory and no‐estimation tasks. However, further investigation is necessary to confirm this hypothesis.

Increased alpha‐band ERD was observed in contralateral frontal regions, both medially and laterally, during hedonic estimations of hessian relative to silk. The OFC has previously been implicated in the processing of a range of un/pleasant stimuli, including scents, words, temperature and touch stimuli (Frey et al., 2009; Grabenhorst et al., 2007; Kringelbach, 2005; Lewis et al., 2007; Rolls, 2010, 2020; Rolls et al., 2008; Rolls, Kringelbach, & de Araujo, 2003; Rolls, O'Doherty, et al., 2003). The observed frontal activation may correspond to the DLPFC, which demonstrates increased activation during estimation and comparison tasks with textured stimuli (Sathian et al., 2011; Simões‐Franklin et al., 2011; Yang et al., 2017) and may reflect the storage of tactile information in working memory to later inform estimation tasks (Barbey et al., 2013; Zhao, Zhou, et al., 2018). Notably, a recent investigation of texture processing during active exploration of surface textures with functional near‐infrared spectroscopy revealed prefrontal activation related to hedonic preference (Marschallek et al., 2023), further demonstrating the critical role of the prefrontal cortex in hedonic processing and estimation of surface texture during active touch.

The use of force plate technology to investigate texture perception during active touch increases ecological validity by allowing participants to optimise their exploratory procedure to gather somatosensory information. This approach also enables accurate data fusion, allowing for the time‐locking of ERD/S to the onset of tactile exploration, the removal of noisy trials with atypical touch behaviour and the recording of touch behaviour such as load, friction and speed (Henderson et al., 2022). Interestingly, load increased for hedonic estimation tasks compared with no‐estimation tasks, suggesting exploratory behaviour varies by task type. Previous research shows that roughness estimates are modulated by exerted force, revealing a link between haptic exploration and perception of surface and object properties (Lederman & Taylor, 1972; Tanaka et al., 2014). Tactile data were also used by implementing covariates on a single‐trial basis to account for variance in touch behaviour and understand the invariant effect of texture and estimation on the neural response; therefore, ERD differences seen between conditions cannot be ascribed to behavioural differences in active touch.

However, the present study is limited in replicating natural tactile experiences because of the use of forearm support and the EEG laboratory setting. Participants were also exposed to the two textured stimuli repeatedly over the testing period, which could lead to sensory desensitisation (Graczyk et al., 2018; Klingner et al., 2011), though repeated trials are necessitated by the time–frequency method (Cohen, 2017). Nevertheless, subjective ratings showed that participants were not desensitised to the textured stimuli per se, but rather sensitised to unpleasant rough stimuli. Furthermore, each block contained all three types of trial, which aimed to maximise participant engagement. However, this choice of task may be limited by the potential influence of prior knowledge from the estimation trials on participants' responses during the no‐estimation trials. Additionally, active touch paradigms inherently increase the likelihood of motor‐related artefacts in EEG data. However, despite this limitation, studying active touch is crucial for enhancing ecological validity and gaining insights into the potential unique neural mechanisms associated with active touch.

This study offers novel insights into texture perception during active touch and highlights the potential to improve ecological validity in future research by using force plate technology. In particular, the current paradigm may be used with neuroimaging methods with higher spatial resolution, such as magnetoencephalography, to explore the involvement of region‐specific processing during hedonic estimation of texture. Additionally, future investigation should consider increasing the number of stimuli; this could be achieved using a gel‐based EEG system, which allows for longer recording times compared with saline‐based EEG systems. Alternatively, employing neuroimaging techniques, such as functional near‐infrared spectroscopy, that require fewer trials for averaging could be explored (Marschallek et al., 2023).

## 5 CONCLUSION

In conclusion, the study found that active exploration of textures had differential impacts on oscillatory brain activity, with rough textures increasing alpha‐band ERD in contralateral sensorimotor regions. Hedonic processing of rough textures elicited increased temporoparietal beta‐band and frontal alpha‐band ERD, indicating selective activation of higher order brain regions for the processing of less preferred stimuli. Future research should continue to explore the neural mechanisms underlying the perception of textures during active touch and their modulation by different modes of stimulation and cognitive tasks.

## AUTHOR CONTRIBUTIONS

**Jessica Henderson**: Conceptualization, Methodology, Software, Formal analysis, Investigation, Data curation, Writing—original draft, Writing—review & editing, Visualisation, Project administration. **Tyler Mari**: Investigation, Writing—review & editing. **Danielle Hewitt**: Conceptualization, Investigation, Writing—review & editing. **Alice Newton‐Fenner**: Investigation, Writing—review & editing. **Andrew Hopkinson**: Methodology, Software, Writing—review & editing. **Timo Giesbrecht**: Conceptualization, Funding acquisition, Supervision. **Alan Marshall**: Conceptualization, Supervision, Writing—review & editing. **Andrej Stancák**: Conceptualization, Supervision, Writing—review & editing. **Nicholas Fallon**: Conceptualization, Methodology, Writing—review & editing, Supervision, Funding acquisition.

## CONFLICT OF INTEREST STATEMENT

None.

### PEER REVIEW

The peer review history for this article is available at https://www.webofscience.com/api/gateway/wos/peer-review/10.1111/ejn.16101.

## Supporting information


**Figure S1.** Exemplary VAS for a sensory estimation trial.
**Figure S2.** The statistical design as implemented in SPM12. (A) Exemplary GLM design matrix for a single subject. Each column represents a model regressor; trials are listed in rows, sorted according to texture and estimation condition. The first six regressors represent binary variables specifying the trials' condition; hedonic hessian (HH), sensory hessian (SH), no estimation hessian (NH), hedonic silk (HS), sensory silk (SS), and no estimation silk (NS). The remaining six regressors were entered as covariates (CV). (B‐F) Exemplary contrast weights to produce contract images to test of the effect of texture; (B) the difference of hessian (SH, HH and NH) vs. silk (SS, HS, and NS). To test the effect of estimation; (C) the difference of sensory (SH and SS) vs. hedonic (HH and HS), and (D) the difference of hedonic (HH and HS) vs no estimation (NH and NS). To test the interaction effect; (E) the difference of sensory hessian and hedonic silk (SH and HS) vs. hedonic hessian and sensory silk (HH and SS), and (F) hedonic hessian and no estimation silk (HH and NS) vs no estimation hessian and hedonic silk (NH and HS).

## Data Availability

The data that support the findings of this study are openly available on Open Science Framework at osf.io/g2dnt.
