# Supplementary material for: Tactile estimation of hedonic and sensory properties during active touch: An electroencephalography study
Source: Eur J Neurosci. 2023 Jul 30;58(6):3412–31. doi: 10.1111/ejn.16101 (PMC10946733; doi:10.1111/ejn.16101)
Supplement: Supplementary file 1 — Figure S1. Exemplary VAS for a sensory estimation trial. Figure S2. The statistical design as implemented in SPM12. (A) Exemplary GLM design matrix for a single subject. Each column represents a model regressor; trials are listed in rows, sorted according to texture and estimation condition. The first six regressors represent binary variables specifying the trials' condition; hedonic hessian (HH), sensory hessian (SH), no estimation hessian (NH), hedonic silk (HS), sensory silk (SS), and no estimation silk (NS). The remaining six regressors were entered as covariates (CV). (B‐F) Exemplary contrast weights to produce contract images to test of the effect of texture; (B) the difference of hessian (SH, HH and NH) vs. silk (SS, HS, and NS). To test the effect of estimation; (C) the difference of sensory (SH and SS) vs. hedonic (HH and HS), and (D) the difference of hedonic (HH and HS) vs no estimation (NH and NS). To test the interaction effect; (E) the difference of sensory hessian and hedonic silk (SH and HS) vs. hedonic hessian and sensory silk (HH and SS), and (F) hedonic hessian and no estimation silk (HH and NS) vs no estimation hessian and hedonic silk (NH and HS). [file EJN-58-3412-s001.docx]

# Supplementary Material 1

## PsychoPy instructions

During this task, you will be exploring textures with your index finger and evaluating them.

At the start of a trial, you will see a white cross indicating you should rest with your index finger stationary on the texture.

A shape will then appear, which will correspond to one of three conditions previously outlined.

A green fixation cross will appear, indicating you should start exploring the texture. Think about the texture properties which correspond to the condition shape during your exploration.

Stop touching the texture when the green cross disappears and keep your finger stationary.

You will rate the texture after sensory and hedonic trials using a slide bar, use your left hand and the mouse to submit your rating.

There will be four blocks, halfway through the block you will be asked to remove your finger from the current texture and switch to the other texture.

## Verbal instructions

### After EEG fitting

On the screen you can see the measurements from the EEG cap. There are 129 channels, and each of them records the electrical activity on your scalp. EEG records any electrical activities, including signals from your brain, as well as other activities like muscle movements. For example, please perform a series of blinks. You will notice that each blink results in a spike in the EEG recording. Now, clench your teeth. You can observe that doing so creates a noisy black period in the EEG recording. This demonstrates the importance of remaining as still as possible during the task. During the task, it is important to focus on the computer screen in front of you and try not to look around the room or move your head. Please try to remain as relaxed as possible, particularly in your shoulders, neck, and jaw. Do you need me to make any adjustments to make you more comfortable?

You will have a short break halfway through each block and a longer break at the end of each block, during which you can move freely. If you need to move during the task, please try to limit your movement to the rating period. If you feel uncomfortable at any time, please inform me by either calling for me or knocking on this wall.

Do you have any questions?

### Before tactile exploration task

During this task, you will be exploring textures within your index finger. You will complete four blocks, each lasting approximately 18 minutes. In each block, you will be instructed to explore either texture A or texture B. Texture A refers to the texture on your left, while texture B refers to the texture on your right.

Halfway through each block, you will be asked to remove your finger from the texture. It is important that you do not touch the force plate during this time, as it will be calibrated by the researcher. After the calibration, the task will prompt you to place your finger back down, this time on the alternative texture.

During each trial, you will see a white cross, which indicates that you should keep your finger still on the texture. Following the white cross, a condition indicator will appear on the screen, providing instructions on what feature to attend to during the exploration period or whether no specific feature requires attention.

For you, the triangle indicates a sensory trial. During these trials, you should focus on the sensory features of the texture. Pay attention to how the texture feels - is it soft, hard, smooth, or rough?

If you see the square, it indicates a hedonic trial. During these trials, you should focus on how the exploration of the texture feels. Consider whether it is pleasant, unpleasant, comfortable, or uncomfortable.

Lastly, if the circle appears, it indicates a trial with no estimation. You do not need to attend to any specific feature.

A green cross will subsequently appear on the screen, indicating that you should begin the exploration task. You are free to explore the texture with your index finger in any way you like. It is important to pay attention to the condition indicator and think about the outlined feature while performing your exploration.

After the exploration period for sensory and hedonic trials, you will be asked to rate your experience on a sliding scale. Please use your left hand and the mouse to submit your rating without removing your right index finger from the texture. You will not be asked to make a rating after no estimation trials.

During the task ensure that only your right intext finger is touching the force plate. Please do not rest your hand or other fingers on the texture.

First, you will complete some practice trials to ensure you are comfortable with the trial setup.

Do you have any questions?

## VAS

**
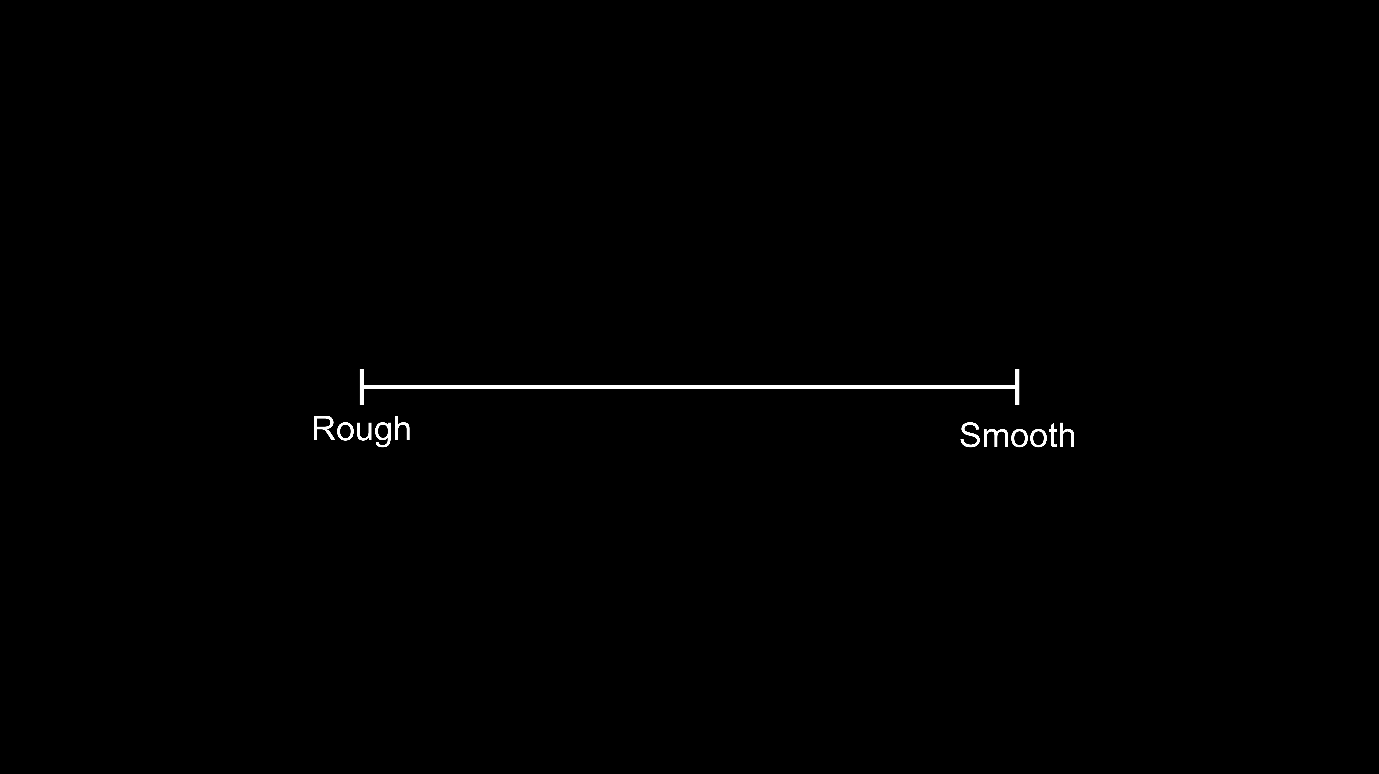
**

Supplementary figure 1. Exemplary VAS for a sensory estimation trial.

# Supplementary Material 2

The statistical design implemented in SPM12 for a single-subject GLM analysis. The design matrix consists of binary variables specifying the condition of the trials and covariates.


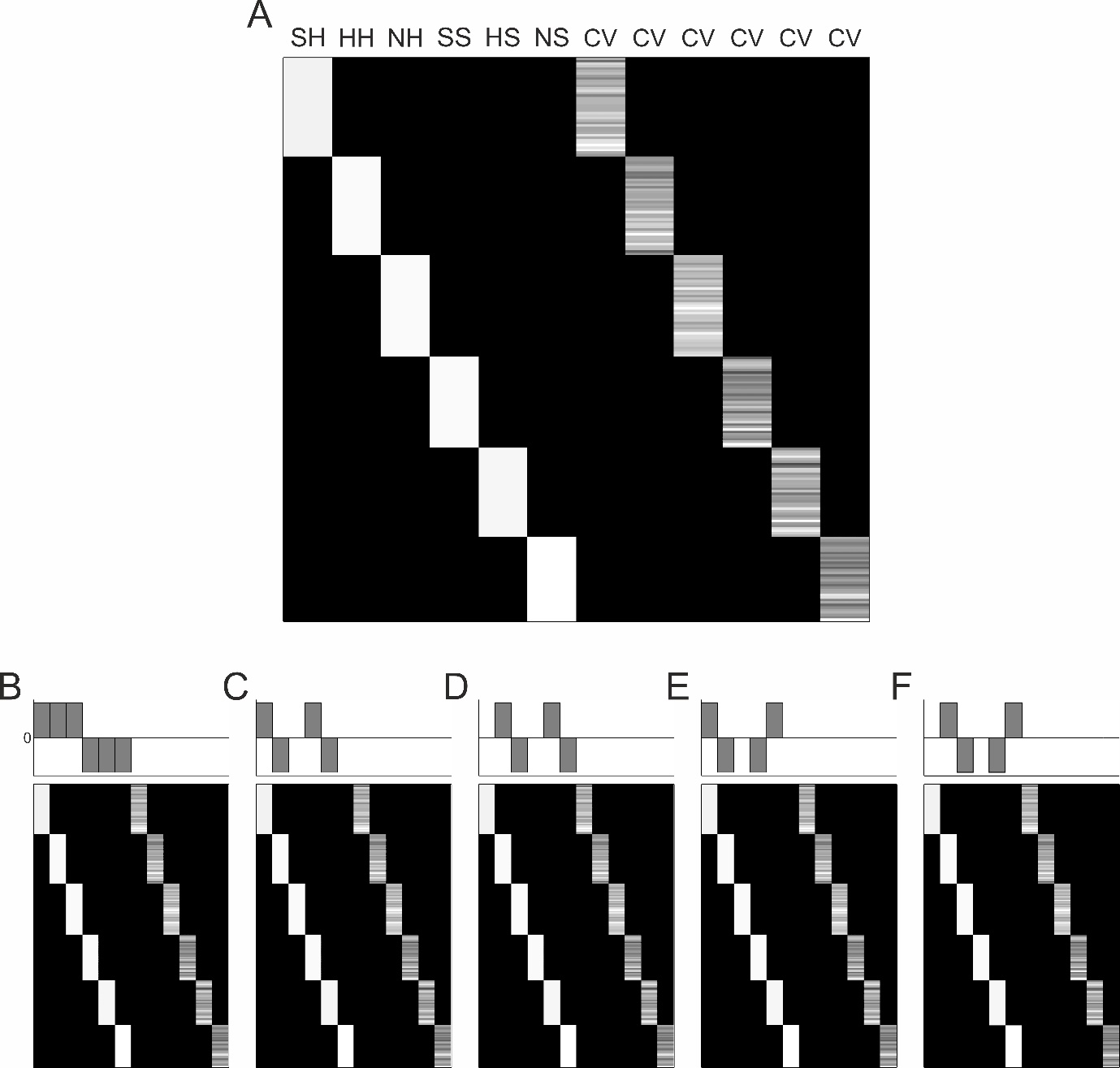


Supplementary figure 2 The statistical design as implemented in SPM12

. (A) Exemplary GLM design matrix for a single subject. Each column represents a model regressor; trials are listed in rows, sorted according to texture and estimation condition. The first six regressors represent binary variables specifying the trials' condition; hedonic hessian (HH), sensory hessian (SH), no estimation hessian (NH), hedonic silk (HS), sensory silk (SS), and no estimation silk (NS). The remaining six regressors were entered as covariates (CV). (B-F) Exemplary contrast weights to produce contract images to test of the effect of texture; (B) the difference of hessian (SH, HH and NH) vs. silk (SS, HS, and NS). To test the effect of estimation; (C) the difference of sensory (SH and SS) vs. hedonic (HH and HS), and (D) the difference of hedonic (HH and HS) vs no estimation (NH and NS). To test the interaction effect; (E) the difference of sensory hessian and hedonic silk (SH and HS) vs. hedonic hessian and sensory silk (HH and SS), and (F) hedonic hessian and no estimation silk (HH and NS) vs no estimation hessian and hedonic silk (NH and HS).
